# Glutathione S-transferases Control astrocyte activation and neuronal health during neuroinflammation

**DOI:** 10.3389/fmolb.2022.1080140

**Published:** 2023-01-06

**Authors:** Ken Matoba, Eisuke Dohi, Eric Y. Choi, Shin-ichi Kano

**Affiliations:** ^1^ Department of Psychiatry and Behavioral Neurobiology, University of Alabama at Birmingham Heersink School of Medicine, Birmingham, AL, United States; ^2^ Department of Psychiatry and Behavioral Sciences, Johns Hopkins University School of Medicine, Baltimore, MD, United States

**Keywords:** astrocyte, glutathione S-transferase, GSTM1, inflammation, neuron, aging

## Abstract

Glutathione S-transferases (GST) are phase II detoxification enzymes of xenobiotic metabolism and readily expressed in the brain. Nevertheless, the current knowledge about their roles in the brain is limited. We have recently discovered that GSTM1 promotes the production of pro-inflammatory mediators by astrocytes and enhances microglial activation during acute brain inflammation. Here we report that GSTM1 significantly affects TNF-α-dependent transcriptional program in astrocytes and modulates neuronal activities and stress during brain inflammation. We have found that a reduced expression of GSTM1 in astrocytes downregulates the expression of pro-inflammatory genes while upregulating the expression of genes involved in interferon responses and fatty acid metabolism. Our data also revealed that GSTM1 reduction in astrocytes increased neuronal stress levels, attenuating neuronal activities during LPS-induced brain inflammation. Furthermore, we found that GSTM1 expression increased in the frontal cortex and hippocampus of aging mice. Thus, this study has further advanced our understanding of the role of Glutathione S-transferases in astrocytes during brain inflammation and paved the way for future studies to determine the critical role of GSTM1 in reactive astrocyte responses in inflammation and aging.

## Introduction

Glutathione *S*-transferases (GSTs) are a family of enzymes that conjugate reduced glutathione (GSH) to target molecules in the phase II detoxification of xenobiotic metabolism (Landi, 2000; Hayes et al., 2005; Petermann et al., 2009). Accumulating evidence shows that GSTs are also involved in a variety of inflammatory and cellular stress conditions (Smeyne et al., 2007; Zhang et al., 2013; Smith et al., 2019). One of the GSTs that was reported to be abundantly expressed in the brain is GSTM1 (Beiswanger et al., 1995; Sharma et al., 2015). We have recently discovered that GSTM1 is selectively expressed in astrocytes in the brain parenchyma and enhances microglial activation during brain inflammation (Kano et al., 2019). Understanding the mechanisms by which GSTM1 controls reactive astrocytes will provide a novel insight into the molecular regulatory mechanisms of reactive astrocytes during brain inflammation and an opportunity to evaluate the roles of GSTM1 and related molecules as potential core mechanisms controlling brain inflammation.

Here we examined the mechanisms of how GSTM1 regulates astrocyte transcriptional program in response to pro-inflammatory signaling and further addressed the impact of GSTM1 loss-of-function on neurons during acute brain inflammation following a systemic injection of lipopolysaccharides (LPS). Our data revealed the broad influence of GSTM1 on the regulation of astrocyte transcriptional programs and implied the requirement of GSTM1 in the maintenance of neuronal activities during brain inflammation.

## Method

### Mice

*mGfap* ^*Cre*^ mice and C57BL/6J mice were purchased from the Jackson Laboratory. C57BL/6 timed pregnant female mice for *in vitro* cell cultures were purchased from Charles River Laboratories or prepared in-house. Mice were housed in specific pathogen-free facilities at Johns Hopkins University and University of Alabama at Birmingham. All the procedures were approved by the Institutional Animal Care and Use Committees at both institutions.

### Lentivirus preparation

Lentiviruses were prepared as described previously (Kano et al., 2019). HEK-293FT cells (Invitrogen) were transfected with pLKO-puro vectors encoding either Gstm1 or control shRNA, and packaging vectors (pMD.2G, psPAX2) by using lipofectamine 3000 (Invitrogen). Then, culture supernatants were collected at 48 h post-transfection and ultracentrifuged 25,000 rpm at 4°C for 2 h to precipitate the virus. Viral pellets were dissolved in PBS and kept frozen at −80°C until use. Virus titers were estimated by using a quantitative PCR (qPCR)-based method to detect LTR sequences as described previously (Kano et al., 2019).

### AAV preparation

AAV-293 cells (Agilent Technologies) were transfected with AAV vectors (pAAV-LStopL-GFP-shRNAmirs) and helper vectors (pAd helper vector and pAAV2/5 packaging vector) following a standard CaCl_2_ method with some modifications (Kano et al., 2019). Transfected cells were harvested 48 h later and lysed by three rounds of freeze-thaw cycles. The viruses were enriched from the cell lysates by OptiPrep^™^ density gradient ultracentrifugation and concentrated using Amicon Ultra-100 K filter unit (Millipore). Virus titers were estimated using a qPCR-based method to detect ITR sequence as described previously (Kano et al., 2019).

### Primary glial cell culture

Primary mouse glial cell cultures were prepared from the cortices of postnatal days 2–5 (P2–5) pups of C57BL/6 mice as described previously (Kano et al., 2019). Cells were suspended in DMEM/F12 supplemented with 15% FBS and penicillin/streptomycin (all from Thermo Fisher Scientific) and seeded onto T-75 flasks (Corning) pre-coated with poly-d-lysine (PDL, 25 μg/ml) at approximately two brains per flask. Medium was changed on day 3, and every 2–3 days thereafter. Lentiviral infection was performed with glial cells on day 10 as described below. To enrich astrocytes, oligodendrocyte lineage cells and microglia on the surface of mixed glial cell culture were vigorously shaken off and astrocytes were then collected as negative fractions after MACS sorting with CD11b Microbeads (Miltenyi Biotec). Then, astrocyte cultures were prepared by seeding 5 × 10^5^ astrocytes, immediately after MACS sorting, onto PDL-coated (10 μg/ml) 6-well plates.

### Lentiviral infection

Mixed glia culture was passaged on day 10 and reseeded at 4–7 × 10^6^ cells onto PDL-coated T-75 flasks. On the next day, lentiviruses were added to the culture at 1:1 MOI (multiplicity of infection). The virus-infected cells were enriched by antibiotics-based selection (puromycin, 2.5 μg/ml) for 72 h beginning at 72 h post-infection. Astrocytes were then enriched as described above for further experiment.

### TNF-α treatment *in vitro* cell stimulation

Enriched primary astrocytes were cultured with or without TNF-α (50 ng/ml) for 6 h. Cells were immediately harvested at the end of 6-h incubation for RNA collection.

### RNA sequencing (RNA-seq) and data analysis

Total RNA was extracted from cultured astrocytes using RNeasy mini kit (Qiagen). Libraries were prepared with NEB Next^®^ Ultra^™^ Directional RNA Library Prep Kit (New England BioLabs) and a 100-bp paired-end sequencing was performed on a HiSeq 2,500 (Illumina) at the Johns Hopkins University (JHU) GRCF core facility. RNA sequencing reads were mapped to the mouse genome (GRCm38 assembly) with STAR (v.2.5.4 b) (Dobin et al., 2013) and transcript levels were quantified using RSEM (v.1.3.0) (Li and Dewey, 2011). Differential gene expression between TNF-α-stimulated and non-stimulated groups was determined using DESeq2 (v.1.20.0) (Love et al., 2014) on R (v.3.5.0). A Benjamini–Hochberg adjusted *p*-value of less than .05 was considered a significant difference. RNA-seq data are available at the NCBI Gene Expression Omnibus under accession number GSE217052.

### Stereotactic surgery and LPS treatment

Mice at 5–6 weeks of age were stereotactically injected with 500 nL of AAV (2.0 × 10^10^ GC/μL) at the rate of 100–200 nL/min into the medial prefrontal cortex (mPFC) bilaterally using a NanoFil syringe (WPI) with a 35G beveled needle. The following stereotactic coordinate was used for injection; AP: + 1.5 mm; ML: ±.2 mm; and DV: −1.8 mm from the bregma. Three to 4 weeks later, LPS (Sigma, O55:B5, 5 mg/kg) was injected intraperitoneally and the brains were harvested 6 h later.

### Immunohistochemistry

Mice were anesthetized with isoflurane and perfused transcardially with ice-cold PBS, followed by 4% paraformaldehyde (PFA). Free-floating coronal sections were prepared at 40 µm in thickness with a Leica cryostat. The sections were then incubated in blocking solution (PBS supplemented with 2% Normal Goat Serum, 1% BSA, 1% Triton-X, .05% Tween-20, and .05% sodium azide) at room temperature for 1 h and incubated at 4°C overnight with the following primary antibodies; rabbit anti-Iba1 (1:400, 019–19741, Wako/FujiFilm), chicken anti-GFP (1:5,000, ab13970, Abcam), mouse anti-NeuN (1:500, MAB377, EMD Millipore), mouse anti-4-hydroxy-2-nonenal (1:100, MHN-020P, JaICA), rabbit anti-c-Fos (1:500, 2250, Cell Signaling). After washing with PBS, the sections were further incubated with fluorophore-conjugated secondary antibodies at 1:400 dilution for 2 h and then stained with DAPI (10 μg/ml) for 10 min at room temperature. The sections were mounted on glass slides with ProLong Diamond antifade mounting medium (Thermo Fisher Scientific). *Z*-stack images were acquired with Zeiss LSM700 and 800 confocal microscopes and a Zen software (Zeiss) at JHU School of Medicine Microscope Facility and UAB Civitan International Research Center (CIRC).

### Image analysis

Maximum intensity projection images were analyzed with ImageJ (NIH). Three independent images from the mPFC area of each mouse stained with anti-Iba1, anti-GFP, anti-NeuN, anti-c-Fos, and anti-4-HNE. (*n* = three to five mice per group). The number of NeuN^+^, c-Fos^+^, and 4-HNE^+^ cells per each visual field was quantified using “Analyze Particles” module. Percentages of c-Fos–positive neurons per total neurons and 4-HNE-positive neurons were also calculated with 20x Z-stack images (*n* = three to four mice for control shRNA and *n* = four to five mice for Gstm1 shRNA).

### Western blotting

Tissue lysates were prepared with radioimmunoprecipitation assay buffer and separated on NuPAGE Bis-Tris Mini Gels (Life Technologies), followed by the transfer to polyvinylidene difluoride membrane (Millipore) following a standard protocol. After blocking in 5% skim milk/phosphate-buffered saline supplemented with .1% Tween 20 (PBS-T), membranes were incubated with the primary antibody overnight at 4°C and then incubated with the secondary antibody for 1 h at room temperature. Gel images were captured using ImageQuant LAS 4000 (GE Healthcare), and the intensities of bands were quantified using Quantity One imaging analysis software (Bio-Rad). The following primary antibodies were used: GSTM1 (1:500; no. 12412-1-AP, Proteintech), and β-actin (1:2000; no. sc-47778, Santa Cruz Biotechnology).

### Statistical analysis

Student’s *t*-test was used and *p* < 0.05 was considered statistically significant. For multiple testing corrections, Tukey’s *post hoc* test, Dunnet’s *post hoc* test, or Sidak’s *post hoc* test was utilized as indicated in Figure legend. All the statistical analysis was performed with GraphPad Prism 8 (GraphPad Software, Inc.).

## Results

### Control of TNF-α-dependent gene expression changes by GSTM1 in cultured astrocytes

**FIGURE 1:**
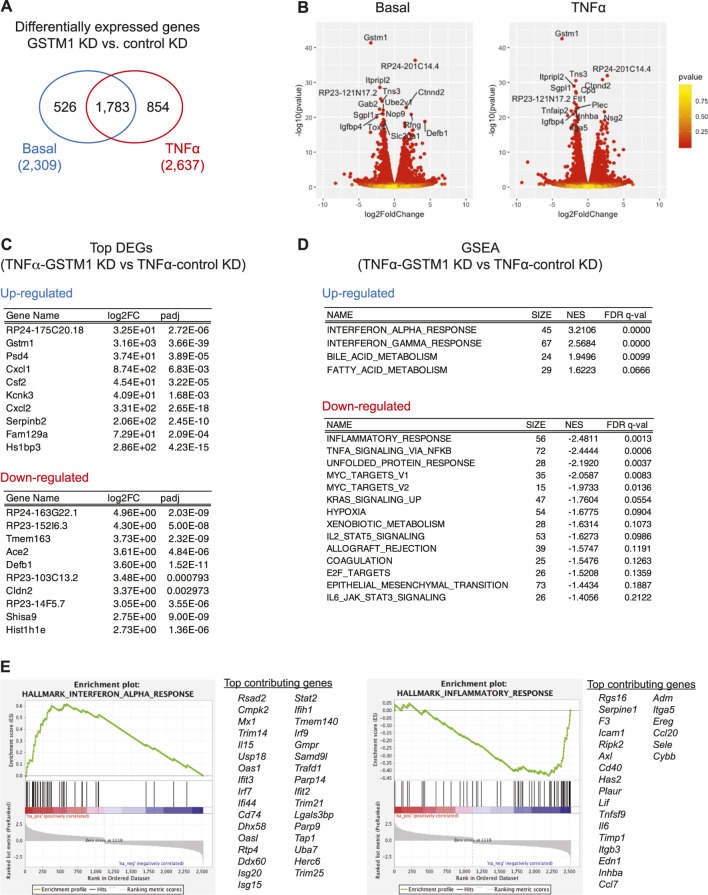
RNA-seq analysis of altered gene expression in cultured astrocytes with reduced GSTM1 expression. Primary mouse cortical astrocytes expressing *Gstm1* or non-silencing control shRNA constructs [GSTM1 knockdown (KD) or control KD] were stimulated with TNF-α (50 ng/ml) for 6 h and their mRNA expression was analyzed by RNA-seq analysis (n = 2 biological replicates per group). **(A)**. Venn diagram of differentially expressed genes (DEGs) in GSTM1 KD or control KD astrocytes. **(B)**. Volcano plots for DEGs between GSTM1 KD and control KD astrocytes with or without TNF-α stimulation. **(C)**. Top DEGs in GSTM1 KD astrocytes in response to TNF-α. **(D)**. Gene set enrichment analysis (GSEA) for DEG in GSTM1 KD astrocytes upon TNF-α stimulation. **(E)**. Representative GSEA plots for interferon alpha responses and inflammatory responses (derived from Molecular Signature database, hallmark gene sets) and their core enrichment genes.

To evaluate the role of GSTM1 in astrocyte activation during acute brain inflammation, we examined the role of GSTM1 in TNF-α-dependent transcriptional program in primary mouse astrocytes. GSTM1 expression was knocked down in astrocyte cultures by infecting them with lentivirus vectors encoding GSTM1 shRNA or non-silencing (NS) controls. Then, GSTM1 knockdown (KD) and control astrocytes were stimulated with TNF-α for 6 h, and their gene expression profiles were analyzed by RNA-seq. A larger number of genes were changed after TNF-α stimulation compared to the basal level (2,637 vs. 2,309) with 1,783 gene expression changes overlapped (Figures 1A, B). We first examined the list of most significantly up/down-regulated genes after TNF-α stimulation (Figure 1C). The mRNA expression of *Gstm1*, but not other *Gstm* genes, dramatically decreased both at the basal level and after TNF-α stimulation in GSTM1 KD astrocytes (Supplementary Table S1). Although we could not completely exclude the possibility that GSTM1 shRNA affect other GST proteins, the data supported that our findings were mainly due to the loss of function of GSTM1. We found that the induction of genes for several cytokines/chemokines, including CXCL1, CSF2, and CXCL2, was severely impaired in GSTM1 KD astrocytes. Among these cytokines and chemokines, CSF2 has been reported to control microglia activity and contribute to neuroprotection in brain inflammation (Ponomarev et al., 2007; Mayo et al., 2014).

We next ran the gene set enrichment analysis (GSEA) for all the 2,637 differentially expressed genes in GSTM1 KD astrocytes after TNF-α stimulation. As shown in Figures 1D, E, the expression of genes related to inflammatory responses and cellular stress responses was significantly downregulated while the expression of genes related to interferon responses and fatty acid metabolism was upregulated (see also Supplementary Table S2). These findings suggest that GSTM1 critically regulates gene transcriptional mechanisms in response to TNF-α stimulation.

### Reduced activities and enhanced cellular stress in the mPFC neurons by GSTM1 knockdown in astrocytes

**FIGURE 2:**
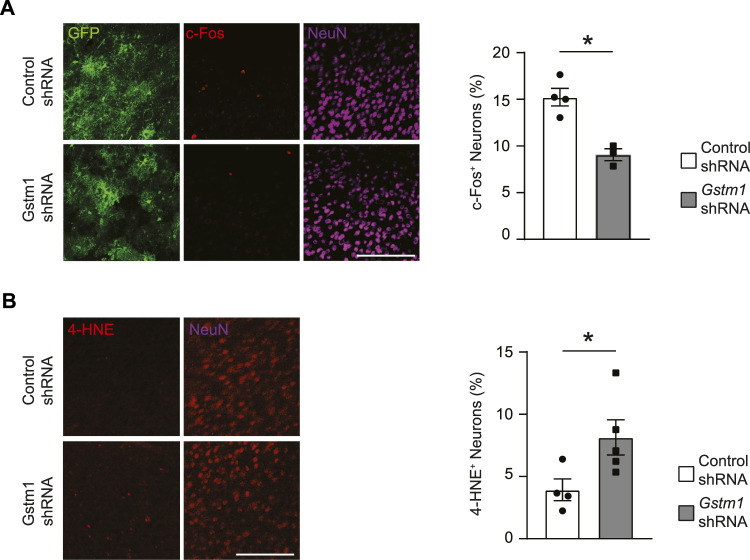
Reduced activities and enhanced cellular stress in neurons associated with GSTM1 KD astrocytes in the brain. Mice with astrocyte-specific expression of *Gstm1* or control shRNA were subject to LPS-induced brain inflammation. The brains were collected at the peak of inflammation (6 h after a systemic LPS injection). c-Fos expression was measured as marker of neuronal activities **(A)** and levels of lipid hyperoxidation (4-HNE staining) were evaluated as cellular stress maker **(B)**. Percentages of c-Fos^+^ NeuN^+^ cells and 4-HNE^+^ NeuN^+^ cells were quantified and compared between *Gstm1* or control shRNA conditions. Graphs show mean ± SEM. Scale bar: 100 µm Control shRNA, *n* = 4; Gstm1 shRNA, *n* = 3–5. Significance was determined by *t*-test. *p < 0.05.

Activation of astrocytes and microglia during brain inflammation affects neuronal health and activities. To determine the impact of reduced GSTM1 expression in astrocytes on neurons, we evaluated the expression of an activity-dependent gene, c-Fos, in neurons of the medial prefrontal cortex (mPFC) during brain inflammation *in vivo*. Mice expressing Cre recombinase under the mouse *Gfap* promoter (*mGfap* ^Cre^ mice) were stereotactically injected with adeno-associated virus (AAV) vectors encoding Gstm1 or non-silencing control shRNAmir into the mPFC as previously described (Kano et al., 2019). Three weeks later, the mice were intraperitoneally administered with lipopolysaccharides (LPS) and the brains were harvested at 6 h. The number of neurons expressing c-Fos was significantly reduced in the area where GSTM1 was knocked down in astrocytes (Figure 2A). Previous studies showed that neuronal activities could enhance intrinsic antioxidant defenses and antiapoptotic activities in neurons (Papadia et al., 2008; Chen et al., 2014). Thus, reduced neuronal activities by GSTM1 KD in astrocytes may result in enhanced cellular stress in neurons. Indeed, the signals of 4-hydroxy-2-nonenal (4-HNE), a marker of oxidative stress, were significantly enhanced in neurons when GSTM1 was reduced in astrocytes (Figure 2B). These findings demonstrate that alterations in GSTM1 KD astrocyte responses to acute brain inflammation negatively impact neuronal activities and increase their cellular stress in a non-cell autonomous manner *in vivo*.

### Aging-associated increase in GSTM1 expression in the brain

**FIGURE 3:**
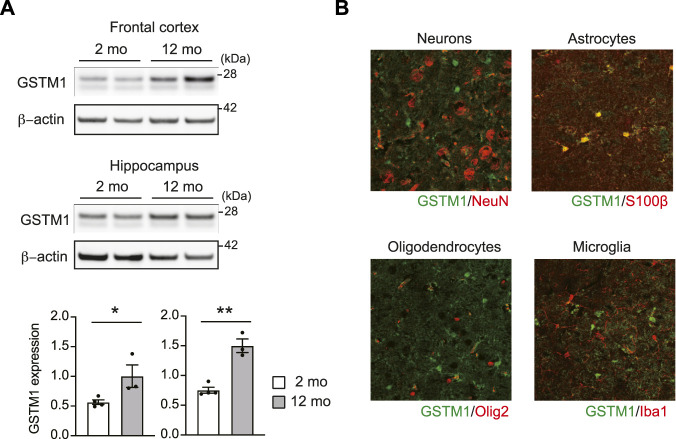
Increased expression of GSTM1 in the aging brains. Western blot for GSTM1 proteins. β-actin was used as a loading control. Quantification of GSTM1 expression relative to β-actin was shown in graphs (mean ± SEM) (*n* = 3-4 per group) **(A)**. Immunohistochemistry of GSTM1 and cell type marker. Scale bar: 50 μm **(B)**. Significance was determined by *t*-test. *p < 0.05, **p < 0.01.

A recent study has reported that the expression level of GSTM1 in the hippocampus is associated with memory ability in 5xFAD mice, which harbors human mutations in the genes for amyloid precursor protein and presenilin one related to early-onset familial Alzheimer’s disease (Neuner et al., 2017). In addition, the expression changes and mutations in multiple GSTs were implicated to increase risks for aging-associated brain disorders associated with inflammation, such as Alzheimer’s disease (AD) (Piacentini et al., 2012; Liu et al., 2015; Wang et al., 2016; Jafarian et al., 2018). Thus, we explored a possible role of GSTM1 in aging mouse brains. We first compared the expression of GSTM1 between young adult (∼2 months old) and middle-aged (∼12 months old) mice. GSTM1 protein expression was increased up to two folds in middle-aged mouse brains compared to young adult brains, at least in the frontal cortex and hippocampus (Figure 3A). Immunohistochemistry data revealed that the expression of GSTM1 protein was mostly enriched for astrocytes in the middle-aged brains similar to our previous observations in the young adult brains (Figure 3B). Aging is associated with increased inflammatory changes and neuronal cellular stress. This data suggests that GSTM1 in astrocytes may play a critical role in the aging brain.

## Discussion

In this brief report, we found that reduced expression GSTM1 in astrocytes decreased the expression of pro-inflammatory genes and enhanced the expression of interferon response genes and fatty acid metabolism-related genes in response to TNF-α stimulation. We further showed that reduced GSTM1 expression in astrocytes impaired the neuronal activities and cellular stress in LPS-induced brain inflammation. These findings have provided a novel mechanistic insight into the role of GSTM1 in astrocytes as a critical modulator of brain homeostasis during inflammatory responses.

How does GSTM1 modulate the expression of a wide variety of genes in response to TNF-α signaling? Our previous study revealed that GSTM1 was required for the optimal activation of NF-κB, a critical transcription factor for the expression of pro-inflammatory genes downstream of TNF-α signaling (Kano et al., 2019). As another GST family enzyme, GSTP, was shown to modulate NF-κB signaling by inhibiting the activities of inhibitory kappa B kinase β (IKKβ), an upstream activator of NF-κB, *via S*-glutathionylation (Jones et al., 2016), GSTM1 may interact with and modulate the upstream regulators of NF-κB. More recently, GSTM1 was shown to bind to and S-glutathionylate TBK1, a kinase required for the activation of interferon regulatory factor 3 (IRF3) critical for the expression interferon genes (Wang et al., 2020). Notably, reduced GSTM1 expression in macrophages enhanced interferon production in macrophage upon virus infection, consistent with our findings. Further mechanistic studies such as the identification of GSTM1 interactomes would determine how GSTM1 differentially impacts NF-κB and IRF transcription factor activities in astrocytes.

GSTM1 in astrocytes may also be involved in aging-associated changes in the brain and possibly aging-associated neurodegenerative diseases. A very recent study reported that a unique aging-associated astrocyte subtype in the hippocampus, exhibiting dysregulated autophagy and morphology, was enriched with GSTM1 expression (Lee et al., 2022). The study further showed that GSTM1 expression gradually increased in the hippocampus during aging. As aging is associated with increased expression of TNF-α and interferons in the brain, these findings indicate that GSTM1 may be actively involved in aging-associated changes in astrocyte inflammatory signaling. Further studies on the impact of GSTM1 deficiency in normal and pathological brain aging may help understand the mechanisms underlying aging-associated brain changes and neurodegeneration.

GSTs are a diverse family of enzymes that defends cells from damage by noxious xenobiotic metabolites (Landi, 2000; Hayes et al., 2005; Petermann et al., 2009). Mice express 22 cytosolic GSTs across eight classes (alpha, mu, pi, sigma, theta, zeta, omega, and kappa) (Hayes et al., 2005). Their expression is observed in various cell types and organs throughout the body. Some GSTs, such as GSTA4, GSTM1, and GSTP1, were detected in the brain (Awasthi et al., 1994; Beiswanger et al., 1995; Al Nimer et al., 2013), but their functions have not yet been fully characterized. Although there are certain homologies in proteins between different types of GSTs, particularly in the same classes, their cell- and tissue-specific expression patterns suggest that they may play distinct roles depending on the context. For example, while GSTP is mostly expressed in mature oligodendrocytes in the mouse brain, their expression is exclusive to neurons in the substantia nigra and controls neuronal sensitivity to MPTP-induced neurodegeneration (Smeyne et al., 2007). We and others have also shown that GSTM1 is enriched in astrocytes and controls astrocyte-mediated inflammatory changes (Sharma et al., 2015; Kano et al., 2019; Lee et al., 2022). Thus, emerging evidence supports for critical roles of GSTs in neuroinflammation and neurodegeneration. As GSTs are similar to each other as proteins, multiple GSTs may function together in specific cell types under a certain conditions. It should be noted that antibodies against most GSTs are not specific and can detect multiple GSTs. Accordingly, in our study, we cannot completely exclude the possibility that *Gstm1* shRNA may alter the expression of other GST proteins. Therefore, further improvements in experimental tools for GST research, such as GST subclass-specific antibodies, are also critical to define the precise role of each GST in neuroinflammation.

As *GSTM1* null genotype is widely observed in humans (Warholm et al., 1981; Rebbeck, 1997; Cotton et al., 2000; Geisler and Olshan, 2001; Josephy, 2010) and has been reported as a risk factor for brain disorders associated with inflammation, such as Alzheimer’s disease (AD) (Piacentini et al., 2012; Liu et al., 2015; Wang et al., 2016; Jafarian et al., 2018), it is tempting to speculate that human GST null genotypes may impact neuroinflammatory responses across individuals, causing severer inflammatory phenotypes in subpopulations of patients with these brain disorders. Nevertheless, we still lack experimental evidence for the role of human GSTs in neuroinflammation. Notably, a recent evolutionary genetic study has reported that the high variability in GSTM genes obscures orthologous relationships between humans and other mammalian species (Tan and Low, 2018). Accordingly, it remains unclear whether GSTM1 or a similarly astrocyte-enriched human GST controls astrocyte responses to inflammatory stimuli, as we observed in mice. Therefore, careful functional characterization of human GSTs in astrocytes under inflammatory conditions is required. With the advance of human induced pluripotent stem cells (iPSC)-based cell and tissue cultures and CRISPR/Cas-based genetic engineering techniques (Hendriks et al., 2020), the impact of human GST genetic mutations on astrocyte function can now feasibly be addressed. Further studies in human GST biology with these new techniques will clarify the significance of GSTs in human neuroinflammation and related brain disorders.

## Data Availability

The datasets presented in this study can be found in online repositories. The names of the repository/repositories and accession number(s) can be found in the article/Supplementary Material.

## References

[B1] Al Nimer F. Strom M. Lindblom R. Aeinehband S. Bellander B. M. Nyengaard J. R. (2013). Naturally occurring variation in the Glutathione-S-Transferase 4 gene determines neurodegeneration after traumatic brain injury. Antioxid. Redox Signal 18, 784–794. 10.1089/ars.2011.4440 22881716 PMC3555113

[B2] Awasthi Y. C. Sharma R. Singhal S. S. (1994). Human glutathione S-transferases. Int. J. Biochem. 26, 295–308. 10.1016/0020-711x(94)90050-7 8187927

[B3] Beiswanger C. M. Diegmann M. H. Novak R. F. Philbert M. A. Graessle T. L. Reuhl K. R. (1995). Developmental changes in the cellular distribution of glutathione and glutathione S-transferases in the murine nervous system. Neurotoxicology 16, 425–440. 8584275

[B4] Chen Z. Jalabi W. Hu W. Park H. J. Gale J. T. Kidd G. J. (2014). Microglial displacement of inhibitory synapses provides neuroprotection in the adult brain. Nat. Commun. 5, 4486. 10.1038/ncomms5486 25047355 PMC4109015

[B5] Cotton S. C. Sharp L. Little J. Brockton N. (2000). Glutathione S-transferase polymorphisms and colorectal cancer: A HuGE review. Am. J. Epidemiol. 151, 7–32. 10.1093/oxfordjournals.aje.a010124 10625170

[B6] Dobin A. Davis C. A. Schlesinger F. Drenkow J. Zaleski C. Jha S. (2013). Star: Ultrafast universal RNA-seq aligner. Bioinformatics 29, 15–21. 10.1093/bioinformatics/bts635 23104886 PMC3530905

[B7] Geisler S. A. Olshan A. F. (2001). GSTM1, GSTT1, and the risk of squamous cell carcinoma of the head and neck: A mini-HuGE review. Am. J. Epidemiol. 154, 95–105. 10.1093/aje/154.2.95 11447041

[B8] Hayes J. D. Flanagan J. U. Jowsey I. R. (2005). Glutathione transferases. Annu. Rev. Pharmacol. Toxicol. 45, 51–88. 10.1146/annurev.pharmtox.45.120403.095857 15822171

[B9] Hendriks D. Clevers H. Artegiani B. (2020). CRISPR-cas tools and their application in genetic engineering of human stem cells and organoids. Cell Stem Cell 27, 705–731. 10.1016/j.stem.2020.10.014 33157047

[B10] Jafarian Z. Saliminejad K. Kamali K. Ohadi M. Kowsari A. Nasehi L. (2018). Association of glutathione S-transferases M1, P1 and T1 variations and risk of late-onset Alzheimer's disease. Neurol. Res. 40, 41–44. 10.1080/01616412.2017.1390902 29072550

[B11] Jones J. T. Qian X. van der Velden J. L. Chia S. B. McMillan D. H. Flemer S. (2016). Glutathione S-transferase pi modulates NF-κB activation and pro-inflammatory responses in lung epithelial cells. Redox Biol. 8, 375–382. 10.1016/j.redox.2016.03.005 27058114 PMC4827796

[B12] Josephy P. D. (2010). Genetic variations in human glutathione transferase enzymes: Significance for pharmacology and toxicology. Hum. Genomics Proteomics 2010, 876940. 10.4061/2010/876940 20981235 PMC2958679

[B13] Kano S. I. Choi E. Y. Dohi E. Agarwal S. Chang D. J. Wilson A. M. (2019). Glutathione S-transferases promote proinflammatory astrocyte-microglia communication during brain inflammation. Sci. Signal 12, eaar2124. 10.1126/scisignal.aar2124 30783009 PMC6637164

[B14] Landi S. (2000). Mammalian class theta GST and differential susceptibility to carcinogens: A review. Mutat. Res. 463, 247–283. 10.1016/s1383-5742(00)00050-8 11018744

[B15] Lee E. Jung Y.-J. Park Y. R. Lim S. Choi Y.-J. Lee S. Y. (2022). A distinct astrocyte subtype in the aging mouse brain characterized by impaired protein homeostasis. Nat. Aging 2, 726–741. 10.1038/s43587-022-00257-1 37118130

[B16] Li B. Dewey C. N. (2011). Rsem: Accurate transcript quantification from RNA-seq data with or without a reference genome. BMC Bioinforma. 12, 323. 10.1186/1471-2105-12-323 PMC3163565 21816040

[B17] Liu H. Peng J. Gao J. Zheng F. Tie C. (2015). Glutathione S-transferase T1 and M1 null genotypes and Parkinson's disease risk: Evidence from an updated meta-analysis. Neurol. Sci. 36, 1559–1565. 10.1007/s10072-015-2159-4 25868597

[B18] Love M. I. Huber W. Anders S. (2014). Moderated estimation of fold change and dispersion for RNA-seq data with DESeq2. Genome Biol. 15, 550. 10.1186/s13059-014-0550-8 25516281 PMC4302049

[B19] Mayo L. Trauger S. A. Blain M. Nadeau M. Patel B. Alvarez J. I. (2014). Regulation of astrocyte activation by glycolipids drives chronic CNS inflammation. Nat. Med. 20, 1147–1156. 10.1038/nm.3681 25216636 PMC4255949

[B20] Neuner S. M. Wilmott L. A. Hoffmann B. R. Mozhui K. Kaczorowski C. C. (2017). Hippocampal proteomics defines pathways associated with memory decline and resilience in normal aging and Alzheimer's disease mouse models. Behav. Brain Res. 322, 288–298. 10.1016/j.bbr.2016.06.002 27265785 PMC5135662

[B21] Papadia S. Soriano F. X. Leveille F. Martel M. A. Dakin K. A. Hansen H. H. (2008). Synaptic NMDA receptor activity boosts intrinsic antioxidant defenses. Nat. Neurosci. 11, 476–487. 10.1038/nn2071 18344994 PMC2556874

[B22] Petermann A. Miene C. Schulz-Raffelt G. Palige K. Holzer J. Glei M. (2009). GSTT2, a phase II gene induced by apple polyphenols, protects colon epithelial cells against genotoxic damage. Mol. Nutr. Food Res. 53, 1245–1253. 10.1002/mnfr.200900110 19753610

[B23] Piacentini S. Polimanti R. Squitti R. Ventriglia M. Cassetta E. Vernieri F. (2012). GSTM1 null genotype as risk factor for late-onset Alzheimer's disease in Italian patients. J. Neurol. Sci. 317, 137–140. 10.1016/j.jns.2012.01.026 22381228

[B24] Ponomarev E. D. Shriver L. P. Maresz K. Pedras-Vasconcelos J. Verthelyi D. Dittel B. N. (2007). GM-CSF production by autoreactive T cells is required for the activation of microglial cells and the onset of experimental autoimmune encephalomyelitis. J. Immunol. 178, 39–48. 10.4049/jimmunol.178.1.39 17182538

[B25] Rebbeck T. R. (1997). Molecular epidemiology of the human glutathione S-transferase genotypes GSTM1 and GSTT1 in cancer susceptibility. Cancer Epidemiol. Biomarkers Prev. 6, 733–743. 9298582

[B26] Sharma K. Schmitt S. Bergner C. G. Tyanova S. Kannaiyan N. Manrique-Hoyos N. (2015). Cell type- and brain region-resolved mouse brain proteome. Nat. Neurosci. 18, 1819–1831. 10.1038/nn.4160 26523646 PMC7116867

[B27] Smeyne M. Boyd J. Raviie Shepherd K. Jiao Y. Pond B. B. Hatler M. (2007). GSTpi expression mediates dopaminergic neuron sensitivity in experimental parkinsonism. Proc. Natl. Acad. Sci. U. S. A. 104, 1977–1982. 10.1073/pnas.0610978104 17267597 PMC1785361

[B28] Smith G. A. Lin T. H. Sheehan A. E. Van der Goes van Naters W. Neukomm L. J. Graves H. K. (2019). Glutathione S-transferase regulates mitochondrial populations in axons through increased glutathione oxidation. Neuron 103, 52–65. 10.1016/j.neuron.2019.04.017 31101394 PMC6616599

[B29] Tan H. M. Low W. Y. (2018). Rapid birth-death evolution and positive selection in detoxification-type glutathione S-transferases in mammals. PLoS One 13, e0209336. 10.1371/journal.pone.0209336 30586459 PMC6306238

[B30] Wang M. Li Y. Lin L. Song G. Deng T. (2016). GSTM1 null genotype and GSTP1 Ile105Val polymorphism are associated with alzheimer's disease: A meta-analysis. Mol. Neurobiol. 53, 1355–1364. 10.1007/s12035-015-9092-7 25633095

[B31] Wang Y. Wang P. Zhang Y. Xu J. Li Z. Li Z. (2020). Decreased expression of the host long-noncoding RNA-GM facilitates viral escape by inhibiting the kinase activity TBK1 via S-glutathionylation. Immunity 53, 1168–1181 e7. 10.1016/j.immuni.2020.11.010 33326766

[B32] Warholm M. Guthenberg C. Mannervik B. von Bahr C. (1981). Purification of a new glutathione S-transferase (transferase mu) from human liver having high activity with benzo(alpha)pyrene-4, 5-oxide. Biochem. Biophys. Res. Commun. 98, 512–519. 10.1016/0006-291x(81)90870-6 7194639

[B33] Zhang B. Gaiteri C. Bodea L. G. Wang Z. McElwee J. Podtelezhnikov A. A. (2013). Integrated systems approach identifies genetic nodes and networks in late-onset Alzheimer's disease. Cell 153, 707–720. 10.1016/j.cell.2013.03.030 23622250 PMC3677161

